# Evaluating Scale-Up Cultivation Modes for *Aspergillus oryzae* Biomass Production Using VFA-Rich Effluents from Agro-Industrial Residues

**DOI:** 10.3390/biotech14040076

**Published:** 2025-09-24

**Authors:** Taner Sar, Clarisse Uwineza, Mohammad J. Taherzadeh, Amir Mahboubi

**Affiliations:** 1 Swedish Centre for Resource Recovery, University of Borås, 50190 Borås, Sweden; st.taner@gmail.com (T.S.); mohammad.taherzadeh@hb.se (M.J.T.); amir.mahboubi_soufiani@hb.se (A.M.); 2 Bioextrax AB, 22478 Lund, Sweden

**Keywords:** *Aspergillus oryzae*, scale-up, volatile fatty acids, cultivation modes, fed batch, fungal biomass composition

## Abstract

Organic-waste-derived volatile fatty acids (VFAs) are promising substrates for fungal biomass cultivation, offering a nutrient-rich medium capable of meeting microbial growth requirements. However, the growth and biomass productivity are highly influenced by the VFAs’ composition and mode of operation. This study investigated the cultivation of *Aspergillus oryzae* fungal biomass using agro-industrial-derived VFA effluent, employing repeated-batch and fed-batch (stepwise and continuous-feeding) cultivation modes to evaluate fungal growth and biomass composition. The highest dry biomass yield of 0.41 dry biomass/gVFAs_fed_ (g/g) was achieved in fed-batch mode with continuous feeding, where the biomass exhibited pellet morphology, facilitating ease of harvesting. The crude protein content varied according to the cultivation strategy, reaching 45–53% in continuous-feeding fed-batch mode, while it was 34–42% in stepwise fed-batch mode. Additionally, the fungal biomass contained significant levels of essential macronutrients and trace elements, including Mg, Ca, K, Mn, and Fe, which are crucial if the biomass is intended to be used in animal feed formulations. This study highlights the effects of cultivation modes on biomass composition and the potential of VFA-derived fungal biomass as a sustainable feed ingredient.

## 1. Introduction

The increasing global population, projected to reach 9.7 billion by 2050 [1], coupled with evolving consumption patterns and a growing demand for both food and animal feed, has placed immense pressure on existing food production systems. Global food demand is anticipated to increase by 70% to 100% by 2050 [2], further propelled by the rising need for animal-based proteins [3]. Satisfying this demand will likely require a 30–60% growth in global crop production for food and feed, potentially leading to unfavorable environmental consequences, including resource use, land and water scarcity, deforestation, and food and feed competitions [4]. Simultaneously, the rapid accumulation of waste, driven by population growth, exceeding two billion tonnes of municipal solid waste annually, a large portion of which is organic, presents a major environmental challenge [5]. In addition, agro-food industries generate tremendous quantities of biodegradable solid or liquid side streams containing processed raw materials’ organic residues [6]. These connected challenges underscore the urgent need for sustainable and integrated solutions that not only enhance food production but also effectively manage organic waste streams.

Anaerobic digestion (AD) offers a versatile and environmentally sustainable approach to waste utilization and resource recovery, surpassing the limitations of conventional techniques like composting, incineration, and landfill disposal. The AD process has the potential to convert complex organic substances into biogas, digestate, and valuable chemical components, particularly volatile fatty acids (VFAs) and hydrogen. VFAs, a mixture of carboxylic acids primarily comprising two to six carbon atoms, including acetic, propionic, butyric, valeric, and caproic acids, are routinely produced during the acidogenesis and acetogenesis phases of AD [7]. The recovered effluent comprises not only VFAs but also essential nutrients, such as nitrogen and minerals. These VFAs hold significant promise as building blocks for production of bioplastics, biofuels, and other high-value products, as well as production of filamentous fungal biomass [8,9,10]. Previous studies have demonstrated that mixed VFAs recovered from the AD of agriculture residues and food waste can be utilized as carbon and nutrient sources for the cultivation of edible filamentous fungal biomass [11,12]. However, the effective utilization of these VFA effluents remain challenging due to the inhibitory effects of acid mixtures as well as variations in acid composition and concentration, which can hinder fungal growth in filamentous fungal cultivation.

Cultivating filamentous fungi offers a promising strategy to address challenges by bioconverting complex organic waste streams and producing protein-rich fungal biomass. Their metabolic versatility and robust enzymatic capabilities facilitate efficient degradation and assimilation of diverse substrates. Notably, *Aspergillus oryzae* stands out as a well-studied and commercially significant filamentous fungi species. Well-known for its historical use in producing traditional fermented foods like shoyu, sake, and miso, its potent enzymatic machinery, including *α*-amylase, *β*-amylase, proteases, and cellulases, effectively contributes to the degradation of complex waste streams [13]. For instance, *A. oryzae* was able to grow well in various waste streams including industrial by-products and VFA-rich effluents [11,14].

Previous investigations into the cultivation of *A. oryzae* in a complex VFA-containing effluent demonstrated fungal growth inhibition, resulting in a prolonged lag phase. This observation suggests substrate inhibition due to a high concentration or complex mixture of VFAs and extended fermentation time due to a long lag phase, which subsequently increases the risk of contamination [11,12,15,16]. This growth inhibition was observed with rising concentrations of both longer-chain VFAs within the mixture and overall VFAs. These findings indicate that although *A. oryzae* possesses the metabolic capacity to utilize VFAs, the efficient conversion of these substrates into fungal biomass is significantly affected by the variable VFA composition and concentration observed across different bio-based effluents. VFA profiles, however, appear to be dependent on a combination of several factors, including the type and composition of organic waste streams and the system’s operating conditions, including the pH and organic loading rate [17,18]. The inherent antimicrobial properties of VFAs, coupled with the variable composition of VFA effluents across diverse waste streams, necessitates tailored cultivation strategies. Batch cultivation, although straightforward and cost-effective, is often limited by substrate inhibition. High initial levels can be toxic to microorganisms, leading to substrate inhibition and suppressed growth [19]. On the other hand, fed-batch cultivation, although susceptible to contamination, helps mitigate substrate inhibition and the effect of initial high concentrations. This approach involves the controlled addition of substrates during the process, optimizing nutrient levels and metabolic conditions, preventing toxicity from high substrate concentrations, and supporting higher biomass and product yields. Various feeding strategies, pulsed, constant, and exponential, are employed, with exponential feeding often delivering superior productivity. Building upon previous research that indicated the effectiveness of a fed-batch approach for inhibitory compounds [20,21], this study hypothesizes that controlled, fed-batch cultivation, implemented within a bubble column bioreactor and designed to gradually introduce VFA-rich effluents to *A. oryzae*, will remediate VFA-mediated inhibitor effects. This attenuation is predicted to shorten the overall cultivation time and enhance the fungal biomass yield, thereby contributing to a more sustainable and efficient bioconversion process.

This study aims to address the challenges of the utilization of VFA-rich effluents derived from the anaerobic digestion of agro-industrial residues like apple pomace and potato protein liquor, as carbon and nutrient sources for scale-up *A. oryzae* cultivation. Based on previous findings [11,12], the VFA composition and concentration in the effluent mixture have an inhibitory effect on fungal growth and reduce overall fungal performance. For this reason, repeated-batch and fed-batch (including stepwise and continuous-feeding) cultivation modes were investigated within a pilot-scale bubble column bioreactor to mitigate VFA-induced inhibition and enhance both biomass production yield and quality.

## 2. Materials and Methods

### 2.1. Substrate Preparation

The VFAs intended for fungal cultivation were produced through the anaerobic co-digestion of apple pomace and potato protein liquor in an immersed membrane bioreactor (iMBR), utilizing rumen fluid as an inoculum, as described by Parchami et al. [22]. The design of the iMBR facilitated the degradation of complex organic compounds and in situ recovery of a particle-free, VFA-rich permeate through microfiltration. The resulting microfiltered VFA effluent, containing nitrogen- and phosphorous-based compounds, minerals, etc., was stored at −18 ± 2 °C until required for fungal growth studies. The chemical composition of the VFA effluent is presented in Table 1.

### 2.2. Fungal Strains

Spores from the filamentous fungus *Aspergillus oryzae* var. *oryzae* CBS 819.72 were used. Stock cultures were maintained on solid Potato Dextrose Agar (PDA) medium, consisting of 4 g/L potato infusion, 20 g/L glucose, and 15 g/L agar. Fresh PDA plates were prepared for spore inoculation. Spores were harvested by adding 20 mL of sterile distilled water to pre-grown cultures, and the resulting suspension was dispersed using a sterile, disposable L-shaped plastic spreader. The inoculated plates were incubated at 30 °C for three days before being stored at 4 °C until further use.

### 2.3. Fungal Cultivation in Pilot-Scale Bubble Column Bioreactor (26 L Capacity)

Experiments were performed in duplicate, employing repeated-batch and fed-batch modes. Fed-batch cultivation was performed in two ways: first, by adding substrates stepwise over time and, second, by supplying the substrate through steady, continuous feeding. Fungal cultivation was carried out in a 26 L bubble column bioreactor (Bioengineering AG, Wald, Switzerland). The bioreactor was sterilized in situ by steam injection at 130 °C for 20 min prior to each experiment.

#### 2.3.1. Repeated-Batch Cultivation

*A. oryzae* seed culture, initially grown in synthetic medium containing 10 g/L glucose and 5 g/L yeast extract in a 1000 mL Erlenmeyer shake flask with a 400 mL working volume, was first used to inoculate 20 L of acetic acid-containing medium for adaptation. The preculture cultivation medium consisted of 3 g/L acetic acid, a salt solution (3.5 g/L KH_2_PO_4_, 0.75 g/L MgSO_4_·7H_2_O, 1.0 g/L CaCl_2_·2H_2_O, and 7.5 g/L (NH_4_)_2_SO_4_), a 6.7 mL/L trace metal solution, and a 1 mL/L vitamin solution prepared according to [23]. The preculture was incubated at 35 °C for 24 h with an aeration rate of 0.5 vvm (volume of air per volume of medium per minute). After incubation, 15 L of culture was harvested and replaced with 15 L of sterile VFA effluent in the bioreactor. The culture medium was then incubated at 35 °C for another 24 h. This process was repeated once more, resulting in a total fungal cultivation time of 96 h. The chemicals used were all purchased from Sigma-Aldrich Co., Ltd., Darmstadt, Germany.

#### 2.3.2. Fed-Batch Cultivation

The experimental design was based on previous findings (under publication) indicating the strong inhibition of *A. oryzae* in batch cultures when butyric acid predominated in the effluent from the co-digestion of apple pomace and potato protein liquor. To mitigate these inhibitory effects and enhance biomass production, a fed-batch cultivation strategy was employed, with feeding intervals of 12 h and 24 h applied to a preculture already adapted to acetic acid. Building on these prior observations, the present study implemented fed-batch cultivation using two different feeding strategies. In the first fed-batch cultivation, *A. oryzae* seed culture, prepared as described in the paragraph above, was used to inoculate 5 L of acetic acid-containing preculture medium (with same composition as described in above paragraph) at 35 °C with an aeration rate of 0.5 vvm (volume of air per volume of medium per minute) for 24 h. After 24 h, 7.5 L of sterile VFA was added to the bioreactor at the 24th h and again at the 48th h, gradually increasing the total volume to 20 L. Fungal cultivation continued for a total of 72 h.

The second fed-batch cultivation involved continuous feeding. Initially, the *A. oryzae* seed culture prepared similarly to the seed culture in batch mode was inoculated into 5 L of acetic acid-containing preculture medium and incubated at 35 °C with an aeration rate of 0.5 vvm (volume of air per volume of medium per minute) for 22 h. After this initial period, sterile VFA effluent was continuously fed into the bioreactor using a peristaltic pump at a rate of 1 L/h for 15 h, and the experiment was stopped at 48 h.

After cultivation, the fungal biomass was harvested, washed with tap water, dried in an oven at 70 °C for 24 h, and stored at room temperature for further analysis.

### 2.4. Analytical Methods

VFA concentrations in fungal growth substrates were determined using gas chromatography with a flame ionization detector (GC-FID). A Perkin-Elmer Clarus 550 gas chromatograph, equipped with an Elite-Wax ETR capillary column (30 m × 0.32 mm × 1.00 µm, Perkin-Elmer, Shelton, CT, USA), was utilized for this analysis. Nitrogen was employed as the carrier gas, maintained at a pressure of 1.38 bar and a flow rate of 2 mL/min. The injector and detector temperatures were set at 250 °C. Prior to injection, samples were prepared as follows: (1) centrifugation at 1200× *g* for 5 min, (2) acidification with a 3:1 (*v/v*) solution of orthophosphoric acid and formic acid, and (3) filtration through a 0.2 µm syringe filter. Butanol was added as an internal standard, and Milli-Q water was used to adjust the final sample volume to 1 mL.

Mineral ion concentrations (sodium, potassium, calcium, iron, magnesium, chromium, manganese, copper, barium, and selenium) within the effluent and the dried fungal biomass were quantified using Microwave Plasma–Atomic Emission Spectroscopy (MP-AES 4200, Agilent Technologies Santa Clara, CA, USA).

Total chemical oxygen demand (COD) and total ammonium nitrogen (NH_4_^+^-N) levels in the media were assayed using Nanocolor^®^ Ammonium analysis kits (MACHERY-NAGEL GmbH & Co., Ltd., KG, Düren, Germany) and a Nanocolor 500D photometer (MACHERY-NAGEL GmbH & Co., Ltd., KG, Düren, Germany).

The Fiber Analyzer A200 (ANKOM Technology, New York, NY, USA) was utilized to assess the acid detergent fiber (ADF) and neutral detergent fiber (NDF), following the instructions provided by the manufacturer.

To determine the fat content, solid samples were subjected to fat extraction using the ST 255 Soxtec extractive system (FOSS, Hillerød, Denmark), with measurements taken according to the supplier’s guidelines.

The crude protein content was determined by applying a factor of 6.25 to the Total Kjeldahl Nitrogen (TKN), which was analyzed using the Kjeldahl method with an InKjel digester and a Behr Test S1 distiller (Behr Labor-Technik, Düsseldorf, Germany). The process began by adding 20 mL of concentrated sulfuric acid (98% H_2_SO_4_, Sigma-Aldrich and Merck, Darmstadt, Germany), KT1 catalyst, and antifoam tablets (Tompson & Capper Ltd., Runcorn, UK) to a pre-weighed 0.25 g dried sample. The mixture underwent digestion for 100 min. Following digestion, the sample was distilled using a unit connected to 50 mL of 4% boric acid (H_3_BO_4_) to collect the resulting vapor. The final step involved titrating the collected solution with 0.1 mol/L hydrochloric acid (HCl, Sigma-Aldrich and Merck, Darmstadt, Germany) to reach a pH of 4.6, with the resulting volume recorded for TKN calculation and crude protein.

The total carbon, nitrogen, and hydrogen content of fungal biomass was quantified utilizing a FlashSmart Elemental Analyzer CHNS/CHNS MVC (Thermo Scientific™ GmbH, Bremen, Germany), which incorporates a dual-furnace configuration and automated sequential operation via MultiValve Control (MVC). This system is connected to a gas chromatographic column that subsequently directs the gas flow to a single channel of the thermal conductivity detector (TCD) for separation and quantification. For sample preparation, 3 to 4 mg of dried biomass was weighed and sealed in tin capsules and subsequently placed on the autosampler (MAS Plus). The samples were then combusted at 950 °C in a quartz reactor under a helium carrier gas flow of 140 mL/min and a pure oxygen combustion gas flow of 250 mL/min.

### 2.5. Statistical Analysis

The statistical significance of the experimental results was determined using data from two independent replicates. The analysis was performed in Minitab 21^®^ (Minitab Ltd., Coventry, UK) using one-way analysis of variance (ANOVA) and Tukey’s test for pairwise comparisons. The result was considered significant if the *p*-value was less than 0.05, corresponding to a 95% confidence level.

## 3. Results and Discussion

### 3.1. Effect of Cultivation Mode on Fungal Biomass Production Using VFAs

**Figure 1:**
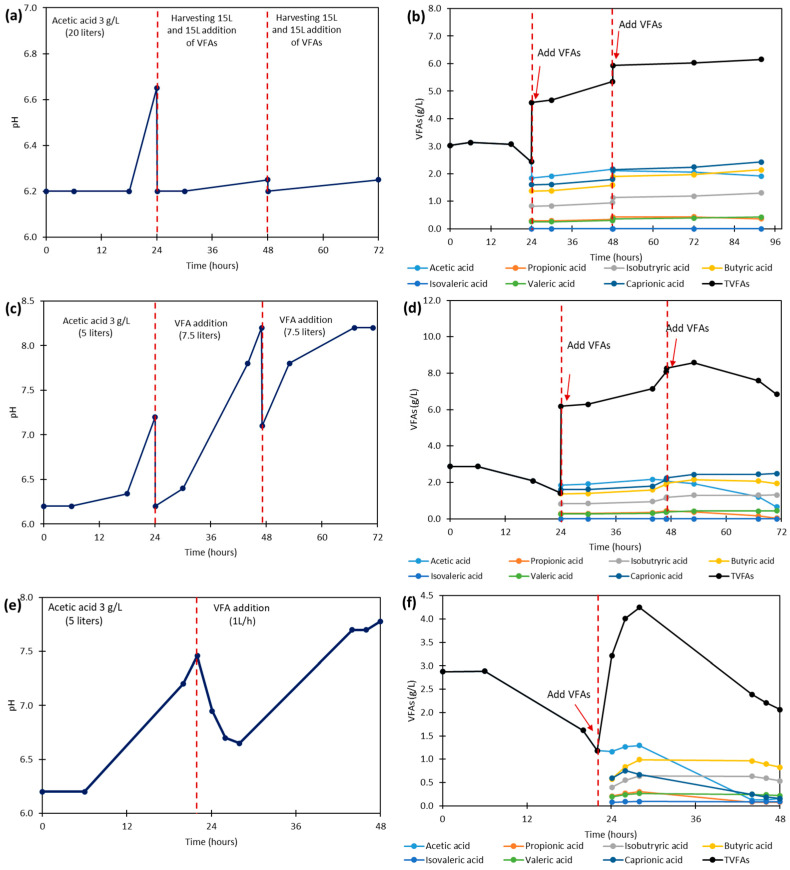
The pH and VFA profiles of *A. oryzae* cultivation during batch (**a**,**b**), stepwise fed-batch (**c**,**d**), and continuous-feeding fed-batch (**e**,**f**) cultivation modes.

A repeated-batch mode was employed to maximize fungal biomass productivity and utilization of VFAs. The rationale of this method was to overcome the significant lag phase identified in prior batch experiments that used a VFA effluent with a comparable total acid concentration [11]. The acquired data on VFA consumption are presented in Figure 1. Contrary to expectations, no fungal growth was observed at 24 h after the first addition, and this inhibition persisted even after the second replacement cycle (Figure 1b). Instead of being metabolized, despite the slight acetic acid consumption, the concentration of total VFAs increased in the medium. This reflects a synergistic interaction among the VFAs, whereby their combined presence amplifies the inhibitory effects on fungal growth and acid utilization, particularly acetic acid, which readily passes through the fungal cell membrane. Mixed-acid conditions generally caused a more pronounced reduction in the growth rate and an extended lag phase compared to individual acids, consistent with a stronger inhibitory effect [24]. Notably, the combination of acetic acid with other VFAs, including propionic, butyric, and caproic acids, markedly delayed acetic acid utilization and intensified the growth inhibition of *A. oryzae* compared to acetic acid alone [11]. The accumulation, along with the absence of a notable pH change (Figure 1a), points to an interruption in metabolic activity and indicates that the acids reached inhibitory concentrations. The observed growth inhibition is inconsistent with previous findings where the fungus demonstrated robust growth on a VFA effluent with similar total acid concentrations [11,12]. This suggests that the specific VFA composition rather than the total concentration was the inhibitory factor, as variations in VFA profiles are known to negatively affect fungal growth. In prior experiments [11,12], the VFA effluents were strongly dominated by acetic acid (50–90%) as the dominant component. In contrast, the VFA effluent in the present study contained a balanced composition with an acetic acid concentration of 23%, comparable to that of longer-chain acids like iso-butyric, butyric, and caproic acids. The presence of these longer-chain acids may have contributed to the observed inhibition, as toxicity generally increases with carbon chain length.

This inhibitory effect can be explained by the difference in the metabolic pathways of the acids. Acetic acid is readily utilized by being directly converted to acetyl-CoA, a central intermediate in many biosynthesis pathways [25]. Conversely, longer-chain acids such as butyrate or caproate cannot be cleaved directly into acetyl-CoA; they require more complex and multiple biochemical transformations before they can enter central metabolism. For instance, butyrate must undergo *β*-oxidation to form butyryl-CoA, which is then converted to acetoacetyl-CoA and subsequently cleaved into acetyl-CoA. These additional metabolic steps make the process less efficient and potentially create inhibitory conditions. Moreover, VFAs with longer carbon chains than acetic acid (C2) (for example, propionic and butyric acid) are known to impair cell membrane integrity and function and disrupt the proton motive force, which causes significant ATP depletion, further stressing the microbial cells and inhibiting growth [26,27]. Consequently, to combat the VFA inhibitory effect and enhance fungal growth and biomass production, an alternative cultivation mode, fed-batch mode, was explored.

Fed-batch mode was used with two different feeding systems, one using stepwise additions of VFA effluents after fungal adaptation and another using continuous feeding. In the stepwise fed-batch mode, 7.5 L of VFA effluent was added at 24 h intervals to a 5 L fungal preculture medium, at 24 h and 48 h (Figure 1c,d). In this method, the fungi gradually consumed acetic acid (54%), while no significant reduction in the other VFAs was observed, resulting in an overall VFA consumption of about 20% (Figure 1d). The produced fungal biomass reached a biomass yield of 0.26 g dry biomass/gVFAs_fed_ (g/g), at 42 h. However, the biomass yield declined to 0.23 g/g at 48 h (*p* = 0.001) and further decreased to 0.14 g/g by 71 h (*p* = 0.000) after the second feeding (Table 2). The poor acid consumption may be attributed to the fact that, prior to VFA addition, the system had not reached stable conditions, which limited fungal adaptation and reduced acid utilization and biomass yield. However, the presence of inhibitory carboxylic acids often results in prolonged fermentation times due to extended lag phases and slower substrate consumption [15]. These extended processes also heighten the risk of contamination, as slow-growing cultures create favorable conditions for competing microorganisms [16]. To address these limitations, fed-batch mode with continuous feeding was employed to mitigate these effects by regulating substrate availability, minimizing inhibition, and accelerating acid utilization and biomass yield.

During fed-batch cultivation with continuous feeding, 15 L of VFA effluent was continuously added to 5 L of fungal preculture, at constant rate of 1 L/h (Figure 1e,f). This method favored both fungal growth and acid utilization, with the fungi consuming approximately 96% of acetic acid, 79% of propionic acid, 90% of caproic acid, and about 40% of other acids, resulting in an overall VFA utilization of 75%. This method yielded better fungal biomass results, with a peak biomass of 2.75 g/L and a yield of 0.41 g/g at 41 h (*p* = 0.000). Similar to the stepwise method, a decline was observed afterward, with the yield decreasing to 0.35 g/g at 46 h (*p* = 0.011) and to 0.22 g/g at 48 h (*p* = 0.001) (Table 2). Although both methods showed a decrease in biomass yield over time, the continuous-feeding method proved more effective, achieving a final biomass yield 1.5 times higher than that achieved through the stepwise fed-batch process.

The decline in the biomass concentration observed, despite an increase in the medium’s pH towards alkalinity over the course of cultivation, suggests the accumulation of inhibitory metabolic by-products, particularly weak carboxylic acids, whose physiological stress is highly pH-dependent. The primary inhibitory mechanism begins when the uncharged, undissociated form of an acid, which predominates at pH levels near or below its pKa (e.g., ~4.75 for acetic acid), passively diffuses across the fungal membrane into the cytosol. In this study, the initial pH was 6.2, and the pH was re-adjusted every 24 h. Inside the near-neutral cytoplasm, the acid dissociates, releasing protons and anions. This leads to cytoplasmic acidification and intracellular accumulation of a less-membrane-permeable anion, both of which are detrimental. To survive these stresses, the fungus must expend a significant amount of energy (ATP) to power membrane pumps that expel the excess protons and restore its internal pH. This critical diversion of ATP away from biosynthesis and cell division is the direct cause of the observed growth inhibition and biomass reduction. This mechanism also explains the experimental findings where growth was reduced despite an increasing medium pH. As acids gradually accumulated during the experiment, the rising external pH did indeed shift the equilibrium towards the dissociated, less permeable form [28]. However, except for acetic acid, the concentration of other acids in the medium remained high. The persistent presence of acids meant that even the small remaining fraction of the toxic undissociated form represented a sufficient and continuous supply to diffuse into the cells, maintaining intracellular stress and suppressing growth despite the increasingly alkaline environment [29]. Furthermore, the fed-batch cultivation with continuous feeding led to a 66% reduction in the total VFA concentration, with decreases of 89%, 79%, and 90% in acetic acid, propionic acid, and caproic acid, respectively (Figure 1f).

**Figure 2:**
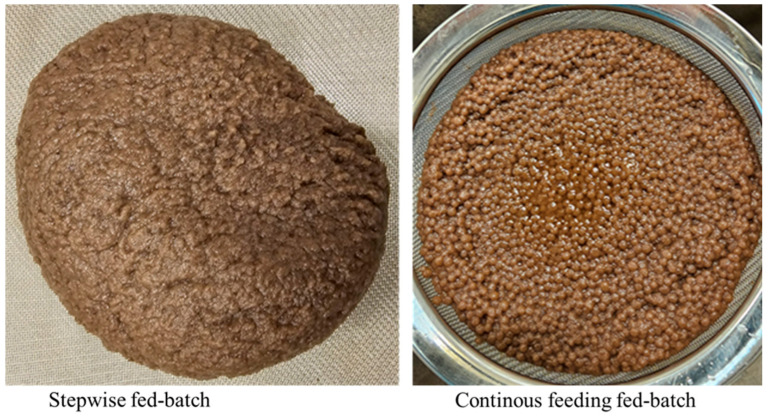
Morphology of *A. oryzae* biomass harvested after stepwise fed-batch (clump) and continuous-feeding fed-batch (pellet) cultivation modes.

Additionally, differences in fungal biomass morphology were observed: biomass grew in clump form during stepwise fed-batch fermentation, whereas it formed pellets in fed-batch mode with continuous feeding (Figure 2). The feeding strategy applied during fermentation strongly influences fungal morphology and broth rheology. In stepwise fed-batch systems, pulsed substrate addition restricts hyphal elongation, resulting in smaller mycelia, reduced viscosity, and improved oxygen transfer. This controlled nutrient supply favors clump formation over pellet development, thereby enhancing mass transfer efficiency. By contrast, continuous feeding maintains constant substrate availability, which promotes pellet formation, larger mycelia, and higher broth viscosity. These conditions hinder oxygen diffusion and can negatively impact overall process performance. Growing fungal biomass in pellet form offers several advantages, including easier biomass harvesting, lower fermentation medium viscosity, improved oxygen diffusion, and higher product yields, particularly for industrial applications requiring high cell density [30].

### 3.2. Effect of Cultivation Mode on Fungal Biomass Composition

The protein content of the fungal biomass obtained in the acetic acid-containing medium (3 g/L) was 55.90%. After VFA feeding in the stepwise fed-batch fermentation, the protein content of the fungal biomass (Table 2) decreased from 42.62% at 48 h to 34.02% at 71 h (*p* = 0.005), representing a 20% decrease in protein content with the extension of the incubation period. Although the protein content of the fungal biomass produced during continuous feeding was significantly higher than that of the biomass produced during stepwise feeding (*p* = 0.039), the protein content of the biomass obtained with continuous VFA feeding decreased by approximately 14% from 53.31% at 41 h to 45.85 at 48 h (*p* = 0.000). This decline in protein content and fungal biomass during extended fungal cultivation is often attributed to nutrient depletion, toxic by-product buildup, or cellular stress leading to cell lysis and endogenous metabolism. In this study, the slower accumulation of VFAs, pH changes, and potentially nutrient depletion suggest biomass degradation and a metabolic shift occurring after a certain time in the fermentation period. Therefore, determining the optimal harvest time is essential to maximize biomass yield and maintain protein quality and content.

Furthermore, the crude protein content observed was consistent with previous studies which reported that the protein content of *A. oryzae* biomass produced from VFAs varies between 34% and 47% [11,12]. *A. oryzae* was grown on other substrates and had a crude protein content of 45.5% in starch processing wastewater [31], 35% in winery wastewater [32] and pomegranate peel [33], and up to 40% in palm oil processing effluents [34]. Additionally, the crude protein levels in soybean and cottonseed meal, which are commercial protein sources for animal feed, range between approximately 40 and 49% and 30 and 50%, respectively [35,36]. These results suggest that the fungal biomass obtained from VFAs in this study may hold promise as a potential protein source.

Although the total fat content of fungal biomass varies depending on the substrate used [37], this study observed differences in fat content even when the same substrate was used in different fermentation modes. The fat content of the biomass was 0.72% in stepwise fed-batch cultivation, whereas it increased to 1.82% in fed-batch cultivation with continuous feeding (Table 3). The differences in substrate accumulation and consumption between these fermentation methods may explain this variation [38]. The primary trigger for lipid accumulation in fungi is the limitation of essential nutrients, mainly nitrogen. When nitrogen is depleted, cell proliferation slows down, but the organism continues to assimilate carbon, redirecting it from biomass production towards lipids synthesis. The efficiency of this conversion, however, depends on the specific VFA’s source and its conversion to acetyl-CoA, the main precursor for lipid synthesis. While acetate is readily converted to acetyl-CoA, other VFAs like propionate and butyrate require more metabolic steps and are assimilated more slowly, potentially inhibiting cell growth [39,40]. These principles explain the observed results. The stepwise fed-batch method led to a transient accumulation of slower-metabolizing VFAs (propionate, butyrate, caproate), which likely constrained the overall rate of lipid synthesis, resulting in a lower final fat content. Conversely, in the fed-batch mode with continuous feeding, the substrate was likely consumed at a rate that led to a gradual depletion of nitrogen as well as the carbon source, acetate, propionate, and caproate, promoting high lipid accumulation.

No significant difference was observed in the acid detergent fiber (ADF) and neutral detergent fiber (NDF) values of fungal biomass obtained using the stepwise fed-batch and continuous fed-batch fermentation technologies (Table 3). Note that the fiber content of the fungal biomass cell wall is in the form of chitin, chitosan, *β*-glucans, and other fungal polysaccharides [41] instead of lignin, cellulose, and hemicellulose. The ADF values ranged from 9% to 11%, representing the proportion of less digestible fiber components, primarily chitin. The NDF values were approximately 25%, indicating the total fiber fraction, including *β*-glucans, chitin, and partially chitosan.

NDF has traditionally been considered an important source of fermentable structural carbohydrates in ruminant diets; however, a higher NDF content does not necessarily imply improved digestibility, as components such as lignin are indigestible and may limit fiber degradation [42]. In contrast, the NDF fraction of fungal biomass is compositionally different from the NDF fraction of plant cell walls, containing mainly chitin, *β*-glucans, and other fungal polysaccharides. Consequently, fungal NDF structural components can provide a readily fermentable and degradable carbohydrate source due to the potential microbial accessibility of these components in the rumen. Previous studies have reported that fungal biomass produced from VFAs contains between 44% and 50% NDF [43]. If fungal biomass is to be used in ruminant feeding, the National Research Council (NRC) recommends a 25% NDF content in the diet of dairy cattle [44], which aligns with the findings of this study. Therefore, filamentous fungal biomass appears to be potentially nutritionally suitable for use as animal feed.

The total nitrogen, carbon, and hydrogen contents of *A. oryzae* biomass varied depending on the fermentation mode and carbon source (Table 4). Biomass obtained from acetic acid-based fermentation had the highest nitrogen content (8.94%), followed by continuous-feeding fed-batch cultivation (8.53% and 6.91%). In contrast, stepwise fed-batch cultivation resulted in lower nitrogen content (4.27% and 5.39%). The higher nitrogen content observed in acetic acid-based and continuous-feeding fed-batch mode suggests a greater proportion of protein-rich biomass, likely due to more efficient nitrogen utilization. The carbon content of *A. oryzae* biomass obtained from VFA-based stepwise fed-batch fermentation ranged from 16% to 19%, while the biomass produced in the continuous-feeding fed-batch fermentation system had a significantly higher carbon content, ranging from 25% to 32%. The higher carbon content observed in the continuous-feeding system and acetic acid-based biomass suggests variations in carbon assimilation efficiency and metabolic pathway regulation under different fermentation conditions.

Apart from the contents of protein and fat, the levels of cell wall components such as chitin, glucan, and mannoproteins, which are carbonaceous compounds known to enhance gut microbiota and feed digestibility, [45,46] may also be analyzed.

The use of volatile fatty acids (VFAs) as a substrate for fungal biomass production created a mineral-rich substrate (Table 5) for microbial growth and biomass composition. The substrate contained high concentrations of essential macronutrients and trace elements, including Na (2.21 g/kg), Mg (11.18 g/kg), Ca (2.45 g/kg), K (12.33 g/kg), and Fe (69.40 g/kg), which are vital for fungal metabolism, enzyme activity, and cell wall synthesis [47]. The minerals with the highest concentration in the fungal biomass were Mg, K, Ca, Na, and Fe (Table 5). The results also indicate that the biomass produced from VFA effluent contains higher levels of Mg, Ca, and Mn compared to biomass derived from acetic acid, likely due to differences in metabolic pathways and environmental conditions that promote enhanced mineral uptake. From a ruminant feed perspective, the biomass obtained from VFA fermentation contains substantial amounts of Mg, which is essential for bone health, enzyme activation, and energy metabolism in animals [48]. Potassium is a major intracellular mineral ion, and when supplemented in animal feeds, it significantly enhances appetite and increases milk production [49]. Calcium plays a role in blood coagulation (vertebrate), muscle functions (such as contractions), nerve impulse transmission, osmoregulation, and hormone and enzyme secretion and serves as a structural component of teeth and bones [50,51].

## 4. Conclusions

This study demonstrated that the choice of cultivation mode is critical when cultivating *A. oryzae* fungal biomass on VFA effluents. Although the repeated-batch mode resulted in growth inhibition due to VFA toxicity, a fed-batch approach with continuous feeding proved to be the most effective at mitigating these effects, achieving the highest biomass yield of 0.41 g/g. This cultivation mode promoted the growth of fungal biomass in a pellet form with a crude protein content of up to 53.31%, alongside higher fat content and elevated levels of minerals like magnesium, potassium, and calcium. Given its nutritional content, the produced fungal biomass shows strong potential as an alternative protein and nutrient source, particularly when used as a substitute in animal feed. In addition, this study suggests that fed-batch cultivation with continuous feeding can effectively overcome VFA-induced inhibition, enhancing both biomass yield and quality.

## Figures and Tables

**Table 1:** Chemical composition of VFA effluent used in the study for fungal cultivation. Results are expressed as mean values ± standard deviation.

Parameters	Units	VFA Effluent
tCOD	g/L	140 ± 13.41
NH4^+^-N	mg/L	450 ± 32.57
tVFAs	g/L	7.99 ± 0.31
Acetic acid	%	23
Propionic acid	%	6
Isobutyric acid	%	15
Butyric acid	%	23
Isovaleric acid	%	1
Valeric acid	%	6
Caproic acid	%	26
pH		6.5
Sodium (Na)	g/L	22.13 ± 0.08
Magnesium (Mg)	g/L	11.18 ± 0.02
Calcium (Ca)	g/L	2.45 ± 0.00
Potassium (K)	g/L	12.33 ± 0.52
Iron (Fe)	g/L	69.40 ± 1.60
Chromium (Cr)	mg/L	17.16 ± 0.45
Manganese (Mn)	mg/L	289.91 ± 8.73
Copper (Cu)	mg/L	24.26 ± 1.15
Barium (Ba)	mg/L	10.23 ± 0.61

**Table 2:** Crude protein contents of fungal biomass obtained from VFAs at different incubation times through fed-batch cultivation with stepwise and continuous feeding. Results are expressed as mean values ± standard deviation.

	Incubation Time (h)	Dry Biomass (g/L)	Biomass Yield (g Dry Biomass/gVFAs_fed_)	Crude Protein (%)
Stepwise	42 h	2.88 ± 0.31	0.27 ± 0.001	Not analyzed
	48 h	2.25 ± 0.22	0.24 ± 0.005	42.62 ± 4.75
	71 h	1.00 ± 0.02	0.15 ± 0.006	34.02 ± 2.91
Continuous	41 h	2.75 ± 0.11	0.41 ± 0.004	53.31 ± 0.29
	46 h	2.36 ± 0.29	0.35 ± 0.000	47.98 ± 0.46
	48 h	1.52 ± 0.18	0.23 ± 0.003	45.85 ± 0.03

**Table 3:** Crude fat, acid detergent fiber (ADF), and neutral detergent fiber (NDF) contents of fungal biomass obtained from VFAs through fed-batch cultivation with stepwise and continuous feeding. Results are expressed as mean values ± standard deviation.

	Incubation Time (h)	Crude Fat (%)	ADF (%)	NDF (%)
Stepwise	48	0.72 ± 0.14	9.08 ± 0.31	25.43 ± 2.93
Continuous	48	1.82 ± 0.14	11.38 ± 1.31	25.80 ± 3.76

**Table 4:** Nitrogen, carbon, and hydrogen content of fungal biomass obtained from acetic acid and VFAs at different incubation times through fed-batch cultivation with stepwise and continuous feeding. Results are expressed as mean values ± standard deviation.

	Incubation Time (h)	Nitrogen (%)	Carbon (%)	Hydrogen (%)
Acetic acid	24	8.94 ± 0.08	44.78 ± 0.10	6.44 ± 0.07
Stepwise	67	4.27 ± 0.54	16.48 ± 3.44	5.00 ± 0.61
	71	5.39 ± 0.18	19.25 ± 0.70	5.81 ± 0.20
Continuous	41	8.53 ± 1.32	32.30 ± 4.12	5.79 ± 1.10
	48	6.91 ± 0.35	25.77 ± 1.44	6.29 ± 0.33

**Table 5:** Mineral profiles of VFAs and fungal biomass obtained from acetic acid and VFAs at different incubation times through fed-batch cultivation with stepwise and continuous feeding. Results are expressed as mean values ± standard deviation.

Minerals	Biomass from Acetic Acid	Stepwise	Continuous
Na (g/kg)	4.64 ± 0.30	3.46 ± 0.37	2.82 ± 0.38
Mg (g/kg)	1.68 ± 0.05	67.74 ± 2.92	42.30 ± 0.13
Ca (g/kg)	0.76 ± 0.01	3.21 ± 0.22	5.39 ± 0.05
K (g/kg)	13.62 ± 0.24	13.56 ± 0.65	10.73 ± 0.39
Fe (g/kg)	4.33 ± 1.07	2.60 ± 0.34	3.20 ± 0.05
Cr (mg/kg)	58.09 ± 2.07	38.88 ± 1.38	43.69 ± 3.28
Mn (mg/kg)	147.79 ± 2.36	415.83 ± 7.15	534.19 ± 0.06
Cu (mg/kg)	987.70 ± 28.31	181.14 ± 8.81	417.14 ± 1.82
Ba (mg/kg)	134.14 ± 2.63	119.75 ± 6.14	148.52 ± 14.35

## Data Availability

The original contributions presented in this study are included in the article. Further inquiries can be directed to the corresponding author.

## Abbreviations

The following abbreviations are used in this manuscript:

AD	Anaerobic digestion
ADF	Acid detergent fiber
Ba	Barium
Ca	Calcium
COD	Chemical oxygen demand
Cr	Chromium
Cu	Copper
Fe	Iron
iMBR	Immersed membrane bioreactor
K	Potassium
Mg	Magnesium
Mn	Manganese
Na	Sodium
NDF	Neutral detergent fiber
NH4^+^-N	Ammonium nitrogen
VFAs	Volatile fatty acids
